# Clinical Remission Trajectories Beyond 12 Months in Severe Asthma Patients Continuing a Single Biologic: A Real-World Single SANI Center Cohort

**DOI:** 10.3390/medsci14050523

**Published:** 2026-08-27

**Authors:** Simone Negrini, Stefania Nicola, Francesco Sardanapoli, Rocco Francesco Rinaldo, Marco Russo, Iuliana Badiu, Anna Quinternetto, Luca Lo Sardo, Francesco Blasi, Pierluigi Paggiaro, Enrico Heffler, Giovanni Rolla, Caterina Bucca, Giorgio Walter Canonica, Fulvia Ribolla, Paolo Solidoro, Luisa Brussino

**Affiliations:** 1 Immunology and Allergy Unit, Azienda Ospedaliera Ordine Mauriziano di Torino, 10128 Turin, Italy; francesco.sardanapoli@unito.it (F.S.); ma.russo@unito.it (M.R.); ibadiu@mauriziano.it (I.B.); aquinternetto@mauriziano.it (A.Q.); llosardo@mauriziano.it (L.L.S.); giovanni.rolla@unito.it (G.R.); caterina.bucca@unito.it (C.B.); luisa.brussino@unito.it (L.B.); 2 Department of Medical Sciences, University of Turin, 10126 Turin, Italy; 3 Pneumology Unit, City of Health and Science, Molinette Hospital, Department of Medical Sciences, University of Turin, 10126 Turin, Italy; roccofrancesco.rinaldo@unito.it (R.F.R.); fulvia.ribolla@gmail.com (F.R.); paolo.solidoro@unito.it (P.S.); 4 Department of Pathophysiology and Transplantation, Università degli Studi di Milano, 20100 Milan, Italy; 5 Respiratory and Cystic Fibrosis Unit, Internal Medicine Department, Fondazione IRCCS Ca’ Granda Ospedale Maggiore Policlinico Milano, 20100 Milan, Italy; 6 Department of Surgery, Medicine, Molecular Biology and Critical Care, University of Pisa, 56100 Pisa, Italy; pierluigi.paggiaro@unipi.it; 7 Personalized Medicine, Asthma and Allergy, Humanitas Clinical and Research Center, Istituto di Ricovero e Cura a Carattere Scientifico (IRCCS), Rozzano, 20089 Milan, Italy; enrico.heffler@hunimed.eu (E.H.); giorgio_walter.canonica@hunimed.eu (G.W.C.); 8 Department of Biomedical Sciences, Humanitas University, Pieve Emanuele, 20072 Milan, Italy

**Keywords:** severe asthma, biologic therapy, clinical remission, type 2 inflammation, oral corticosteroids, real-world evidence

## Abstract

Background: Clinical remission is an emerging therapeutic goal in severe asthma treated with biologics. However, remission may evolve beyond the conventional 12-month assessment, and limited real-world data are available on patients who continue the same biologic after incomplete remission at 12 months. We evaluated longitudinal remission trajectories in patients with severe T2-high asthma continuing the same biologic therapy. Methods: This retrospective real-world single-center SANI cohort included adults with severe asthma treated with one biologic agent and followed for at least 18 months. Clinical outcomes and remission status were assessed at 12 and 18 months according to the SANI definition, distinguishing complete remission, partial remission, and no remission. Results: The cohort included 146 patients; 64.4% were female, median age was 61 years, and all had a T2-high phenotype. Maintenance oral corticosteroid use decreased from 83.6% at baseline to 6.8% at 18 months, while symptomatic patients decreased from 100.0% to 11.0%. Median ACT improved from 12.0 to 22.0, and patients with ≥1 exacerbation decreased to 4.1% at 18 months. Complete remission increased from 36.3% at 12 months to 67.1% at 18 months, while no remission decreased from 54.1% to 16.4%. Among patients not in remission at 12 months, 43.0% achieved complete remission and 26.6% achieved partial remission at 18 months. Baseline oral corticosteroid use was associated with lower remission likelihood, whereas preserved baseline FEV_1_ favored remission at 18 months. Conclusions: Among patients with severe T2-high asthma selected for continuing an unchanged biologic for at least 18 months, remission status was more favorable at 18 months than at 12 months. This observation describes the trajectory of biologic continuers and should not be generalized to all patients who remain outside remission at 12 months. Because patients who discontinued or switched biologics were excluded, these data do not establish that continuation is preferable to switching after an incomplete 12-month response.

## 1. Introduction

Asthma remains a major global health problem, affecting around 300 million people worldwide and imposing a substantial clinical, social, and economic burden on patients, families, healthcare systems, and society [1]. Although severe asthma accounts for only a minority of asthma cases, commonly estimated at 5–10%, it is responsible for a disproportionate share of asthma-related morbidity, healthcare resource utilization, and costs [1,2]. Sex-related differences may further contribute to variability in asthma prevalence, severity, and treatment response, particularly in adult populations [3]. This burden is mainly driven by persistent symptoms, recurrent exacerbations, impaired quality of life, reduced work productivity, repeated exposure to systemic corticosteroids, and the pharmacoeconomic consequences of corticosteroid-related adverse events [4,5,6].

Exacerbations represent one of the most clinically relevant outcomes in severe asthma. They may occur despite apparently acceptable symptom control and are often triggered by viral infections, allergen exposure, air pollution, poor adherence, or relevant comorbidities [1,7]. Real-world data also emphasize that asthma exacerbations are associated with substantial healthcare utilization [8]. Environmental exposures, particularly outdoor air pollution, may contribute to airway inflammation and asthma burden, supporting the relevance of environmental triggers in the long-term course of airway diseases [9]. Beyond acute clinical deterioration, recurrent or severe exacerbations may contribute to cumulative airway injury, repeated treatment escalation, systemic corticosteroid exposure, and accelerated lung function decline, thereby influencing the long-term trajectory of severe asthma and increasing the risk of persistent airflow limitation [10,11].

In recent years, therapeutic goals in severe asthma have evolved from disease control alone toward more ambitious composite outcomes, including clinical remission [12,13]. Biologic therapies targeting IgE, IL-5/IL-5 receptor, IL-4 receptor alpha, and TSLP have consistently reduced exacerbations, improved asthma control and lung function, and decreased maintenance oral corticosteroid dependence in appropriately selected patients [2,5,14,15]. Real-life studies have confirmed clinically meaningful benefits across specific biologic classes and patient phenotypes, including dupilumab effectiveness in severe allergic asthma and improvements in functional, inflammatory, and patient-reported outcomes across different disease profiles [16,17]. In addition, the availability of anti-TSLP therapy has expanded the therapeutic landscape, with real-life data describing upper and lower airway responses to tezepelumab in patients with and without comorbid nasal polyposis [18]. Nevertheless, remission is not achieved in all treated individuals. Real-world data suggest that approximately one third of patients with severe T2-high asthma achieve clinical remission after 12 months of biologic therapy, while longer follow-up may show increasing or more stable remission rates in selected cohorts [19,20,21,22].

The Severe Asthma Network Italy (SANI) has proposed a pragmatic definition of clinical remission based on oral corticosteroid discontinuation, absence of exacerbations, good symptom control, and stable lung function, maintained for one year [23,24]. Complete remission requires fulfillment of all domains, whereas partial remission requires oral corticosteroid discontinuation plus two of the three major clinical domains [23,24]. Although this framework supports standardized assessment, heterogeneity among remission definitions persists [23,24,25]. Remission is usually evaluated after 12 months of biologic therapy; however, treatment response may evolve over time, and some patients may require longer exposure before achieving full clinical benefit. Evidence remains limited on patients who do not meet remission criteria at 12 months but continue the same biologic treatment. Therefore, this study aimed to describe longitudinal remission trajectories between 12 and 18 months among patients with severe T2-high asthma who continued the same biologic therapy after the conventional 12-month assessment [26].

## 2. Materials and Methods

### 2.1. Study Design and Setting

This was a retrospective, observational, single-center real-world study conducted at the Allergy and Clinical Immunology Unit of Mauriziano Hospital, Turin, Italy, which is one of the reference centers for the Severe Asthma Network Italy (SANI). Adult outpatients with severe asthma treated with a single biologic therapy and followed between January 2017 and September 2024 were retrospectively evaluated.

The study was performed in accordance with the principles of the Declaration of Helsinki and Good Clinical Practice guidelines. All patients provided written informed consent for the use of their anonymized clinical data for research purposes. The study was approved by the local Ethics Committee “Area Vasta Nord-Ovest Toscana” (protocol number 73714; study number 1245/2016, approved on 7 December 2016).

### 2.2. Study Population

Patients were eligible if they were aged 18 years or older, had a confirmed diagnosis of severe asthma according to international recommendations [1], had received treatment with a single biologic agent without switching during the observation period, and had at least 18 months of available follow-up while continuing the same biologic.

Patients were excluded for lack of written informed consent, loss to follow-up, pregnancy or breastfeeding, poor adherence to scheduled visits, inability to perform reliable lung function testing, or at least one biologic switch for any reason during the study period.

### 2.3. Clinical Assessment and Follow-Up

All patients were managed in a dedicated severe asthma outpatient clinic and were routinely assessed every 3 months as part of standard clinical care. At each visit, asthma symptoms, treatment adherence, exacerbation history, use of oral corticosteroids, lung function, biomarkers of type 2 inflammation, and patient-reported asthma control were systematically recorded. The same SANI domains and operational criteria were applied at both the 12- and 18-month landmarks. Remission status at each landmark was assigned using the longitudinal clinical information available up to that assessment, including maintenance OCS requirement, symptoms and asthma control, exacerbation history during the relevant assessment period, and longitudinal spirometric stability.

In addition to scheduled visits, patients experiencing an asthma exacerbation were re-evaluated in the outpatient clinic within 7 days from the acute event, in order to document clinical severity, treatment escalation, recovery, and subsequent therapeutic needs.

### 2.4. Data Collection

Demographic and clinical data were extracted from medical records. The following variables were collected: age, sex, body mass index (BMI), smoking status, asthma phenotype, comorbidities, maintenance OCS use, asthma symptoms, exacerbation history, ACT score, lung function, FeNO, blood eosinophil count, serum total IgE, SNOT-22, nasal polyp score, and biologic treatment. Given the potential impact of respiratory multimorbidity on clinical outcomes, COPD/ACOS and ABPA were specifically recorded [27,28]. Moreover, the HES/EGPA group included patients with a previous diagnosis of HES or EGPA whose disease was in remission, although asthma remained uncontrolled.

Baseline serum total IgE levels were recorded before biologic treatment initiation. Peripheral blood eosinophil count, forced expiratory volume in the first second (FEV_1_), fractional exhaled nitric oxide (FeNO), and Asthma Control Test (ACT) score were collected at biologic initiation and at follow-up visits. These biomarkers were interpreted as complementary indicators of type 2 inflammation and biologic eligibility rather than as standalone markers of remission probability [29].

### 2.5. Lung Function Assessment

Pulmonary function tests were performed using the Baires System spirometer (Biomedin, Padua, Italy), in accordance with international standards. At least three acceptable flow–volume curves were obtained, with vital capacity values differing by no more than 5%.

Pre-bronchodilator vital capacity, FEV_1_, and FEV_1_/vital capacity (VC) ratio were recorded and expressed as percentages of predicted values. Bronchodilator reversibility was assessed according to GINA criteria and was considered significant in the presence of an increase in FEV_1_ of at least 200 mL and 12% from baseline after bronchodilator administration [1].

### 2.6. Fractional Exhaled Nitric Oxide

FeNO was measured before spirometry, following standardized American Thoracic Society/European Respiratory Society recommendations [30]. Measurements were performed using a chemiluminescence analyzer (Sievers NOA 280; Sievers, Boulder, CO, USA), calibrated with a certified nitric oxide gas mixture before testing. FeNO was assessed at a constant expiratory flow rate of 50 mL/s and expressed in parts per billion (ppb) [30]. The clinical interpretation of FeNO as a non-invasive marker of type 2 airway inflammation was based on ATS guidance [31].

### 2.7. Laboratory Assessments

Routine blood tests, including complete blood count with peripheral eosinophil count, were obtained at each clinical evaluation. Serum total IgE levels were measured using fluorescent enzyme immunoassays (FEIA; Thermo Fisher Scientific, Waltham, MA, USA), according to the manufacturer’s instructions. Blood eosinophils, FeNO, and IgE were considered among the routinely used biomarkers for asthma phenotyping and biologic eligibility assessment [14,29,32].

### 2.8. Asthma Exacerbations

Asthma exacerbations were defined according to the ATS/ERS statement as acute or subacute worsening of respiratory symptoms associated with airflow limitation and requiring unscheduled medical evaluation and/or treatment intensification [33]. Treatment escalation could include increased controller therapy, oral corticosteroids, antibiotics when clinically indicated, emergency department access, or hospitalization.

Exacerbations were recorded as the number of events occurring between two consecutive scheduled visits and were reported as annualized asthma exacerbation rate.

### 2.9. Asthma Control Assessment

Asthma control was evaluated using the Asthma Control Test at each follow-up visit [34]. An ACT score of 20 or higher was considered indicative of well-controlled asthma, whereas a score below 20 was interpreted as uncontrolled or poorly controlled disease and prompted clinical reassessment and treatment optimization.

### 2.10. Asthma Remission Assessment

Asthma remission was evaluated according to the Severe Asthma Network Italy (SANI) clinical remission framework [23,24]. Complete clinical remission was defined as the simultaneous fulfillment of all four SANI domains: no further need for oral corticosteroids, absence of asthma symptoms, absence of asthma exacerbations or attacks, and stable lung function. Partial clinical remission was defined as no further need for oral corticosteroids plus fulfillment of at least two of the three remaining domains: absence of asthma symptoms, absence of asthma exacerbations or attacks, and stable lung function. Patients who did not meet criteria for complete or partial remission were classified as not in remission.

In accordance with the SANI framework, the oral corticosteroid domain was defined as absence of maintenance oral corticosteroid requirement; the symptom domain was evaluated through clinical assessment and asthma control, including ACT; the exacerbation domain was defined as absence of asthma exacerbations or attacks during the relevant assessment period; and the lung-function domain was defined as stable pulmonary function on longitudinal spirometric assessment. No additional numerical FEV1 cut-off beyond the SANI definition was applied.

Remission status was assessed at 12 and 18 months in patients continuing the same biologic therapy, using the same SANI domains and operational criteria at both landmarks. Remission classification was available for all 146 patients at both 12 and 18 months; therefore, the transition analysis included the entire cohort.

### 2.11. Statistical Analysis

Statistical analysis was performed using a two-sided significance level of 0.05. Demographic, clinical, functional, and biological characteristics of the study population were summarized using descriptive statistics. Continuous variables were reported as mean ± standard deviation or median and interquartile range, according to data distribution, while categorical variables were expressed as absolute numbers and percentages. The distribution of continuous variables was visually inspected and assessed before selecting the appropriate statistical test.

Baseline characteristics included age, sex, body mass index, smoking status, asthma duration, comorbidities, allergic status, asthma phenotype, biologic treatment, lung function, biomarkers of type 2 inflammation, asthma control, oral corticosteroid use, and annualized asthma exacerbation rate.

Longitudinal changes in clinical, functional, and inflammatory outcomes were assessed by comparing baseline values with follow-up evaluations at 12 and 18 months, when available. For paired continuous variables, the paired Student’s *t*-test or Wilcoxon signed-rank test was used, as appropriate. For categorical paired variables, McNemar’s test or Bowker’s test of symmetry was applied when suitable. For repeated dichotomous outcomes across multiple time points, Cochran’s Q test was used. Between-group comparisons were performed using Student’s *t*-test or Mann–Whitney U test for continuous variables, and the chi-square test or Fisher’s exact test for categorical variables, depending on sample size and expected frequencies.

Asthma remission was assessed at 12 and 18 months according to the SANI definition and classified into three mutually exclusive categories: complete remission, partial remission, and no remission. Remission rates were reported as absolute numbers and percentages of evaluable patients.

Changes in remission status between 12 and 18 months were evaluated using a full 3 × 3 transition matrix, quantifying maintenance, improvement, and worsening across complete remission, partial remission, and no remission. For paired binary contrasts (complete remission versus all other states, and any remission versus no remission), exact McNemar tests were used. Absolute paired changes in remission proportions are reported with 95% confidence intervals. These trajectories were also graphically represented using a Sankey diagram.

To explore factors associated with disease remission, patients achieving remission were compared with those not achieving remission at follow-up. Complete and partial remission were analyzed both separately and, when clinically appropriate, together as an overall remission outcome. Candidate predictors included demographic variables, smoking status, body mass index, asthma duration, allergic status, type 2 inflammatory profile, comorbidities, baseline lung function, blood eosinophil count, FeNO, serum total IgE, baseline ACT score, exacerbation history, oral corticosteroid exposure, and biologic class.

Associations between baseline variables and remission outcomes were assessed using prespecified exploratory univariable analyses. We deliberately did not fit a multivariable model because only 24 patients remained outside remission at 18 months, several categorical contrasts contained sparse cells, and baseline OCS exposure produced complete or near-complete separation. In addition, baseline OCS exposure and lung function are conceptually close to components of the remission definition, and biologic allocation was phenotype- and comorbidity-driven rather than randomized, making an adjusted treatment-effect estimate particularly vulnerable to confounding by indication and overfitting. Concomitant controller treatment was essentially uniform across the cohort (high-dose ICS/LABA plus LAMA). Accordingly, the reported associations are hypothesis-generating and should not be interpreted as independent causal predictors or comparative biologic effects.

In analyses involving categorical predictors with sparse cells or complete separation, standard logistic regression estimates were considered unstable; therefore, exact methods or descriptive summaries were used, and these findings were interpreted as exploratory. Analyses involving lung function and biomarkers were performed on available cases only.

All analyses were conducted on available cases, without imputation. Remission category and the principal categorical remission-component indicators (maintenance OCS use, symptom status, and exacerbation status) were available for all 146 patients at both 12 and 18 months. Missingness, when present, was confined to selected continuous spirometric or biomarker measurements; analyses involving these variables used the available denominator. A *p* value < 0.05 was considered statistically significant.

## 3. Results

### 3.1. Cohort Characteristics

The study cohort included 146 patients with severe asthma treated with biologic therapy. Median age was 61.0 (52.0–70.0) years (mean 59.9 ± 13.4), and 94/146 (64.4%) were female. Mean BMI was 24.2 ± 2.3 kg/m^2^, with a median of 23.8 (22.3–26.3) kg/m^2^. Most patients were never-smokers (101/146, 69.2%), whereas 31/146 (21.2%) were former smokers and 14/146 (9.6%) were current smokers. At least one comorbidity was recorded in 69/146 patients (47.3%). CRSwNP was the most frequent comorbidity (55/146, 37.7%), followed by HES/EGPA (9/146, 6.2%) and ABPA (5/146, 3.4%), as shown in Table 1.

### 3.2. Inflammatory Phenotype and Baseline Biomarkers

Using a pragmatic biomarker-based definition (blood eosinophils ≥ 150 cells/uL and/or FeNO ≥ 20 ppb), all evaluable patients fulfilled criteria for a T2-high phenotype (146/146, 100.0%). Baseline FeNO was 47.4 (40.7–53.4) ppb, total IgE was 257.4 (155.9–342.4) IU/mL, and blood eosinophil count was 288 (206–372) cells/uL.

### 3.3. Inhaled and Biologic Therapy

All patients were treated according to GINA guidelines with high-dose ICS/LABA and LAMA throughout the study period. The biologics used in the cohort were Omalizumab: 41/146 (28.1%); Benralizumab: 34/146 (23.3%); Mepolizumab: 28/146 (19.2%); Dupilumab: 41/146 (28.1%); Tezepelumab: 2/146 (1.4%). Biologic treatments are listed in Table 2. Percentages for tezepelumab should be interpreted cautiously because only two patients were included in this subgroup.

Descriptive stratification by biologic showed clinically relevant baseline heterogeneity across treatment groups. Among the four adequately represented biologics, maintenance OCS use ranged from 73.5% with benralizumab to 92.9% with mepolizumab, whereas CRSwNP ranged from 17.1% with omalizumab and 17.9% with mepolizumab to 75.6% with dupilumab. Median baseline FeNO and blood eosinophil counts were broadly overlapping across the main treatment groups. These imbalances are consistent with phenotype- and comorbidity-driven biologic selection in routine practice and create substantial confounding by indication; therefore, treatment-specific differences are presented descriptively and are not interpreted as head-to-head comparative effectiveness estimates. Tezepelumab was not considered suitable for subgroup comparison because only two patients received this agent.

### 3.4. Clinical Outcomes over Follow-Up

Before biologic initiation, 122/146 patients (83.6%) were receiving maintenance oral corticosteroids (OCS), and all patients had persistent asthma symptoms. OCS use decreased progressively to 66/146 (45.2%) at 6 months, 19/146 (13.0%) at 12 months, and 10/146 (6.8%) at 18 months (Cochran Q *p* < 0.001). Similarly, the proportion of symptomatic patients declined from 146/146 (100.0%) at baseline to 86/146 (58.9%) at 6 months, 42/146 (28.8%) at 12 months, and 16/146 (11.0%) at 18 months (Cochran Q *p* < 0.001) (Table 3).

Asthma control improved substantially during follow-up. The median ACT score increased from 12.0 (IQR, 9.0–16.0) at baseline to 16.0 (IQR, 11.2–21.0) at 6 months, 21.0 (IQR, 18.0–22.0) at 12 months, and 22.0 (IQR, 20.0–23.0) at 18 months (Friedman *p* < 0.001). Among paired observations, the median ACT change from baseline to 18 months was 9.0 points (IQR, 5.0–13.0; Wilcoxon *p* < 0.001).

All the patients had at least two exacerbations in the 12-month period before biologic initiation. During follow-up, the proportion of patients experiencing at least one exacerbation decreased from 46/146 (31.5%) at 6 months to 14/146 (9.6%) at 12 months and 6/146 (4.1%) at 18 months (Cochran Q *p* < 0.001).

Median FEV1 was 2.3 L (IQR, 2.0–2.5) at baseline, 2.1 L (IQR, 1.4–2.3) at 6 months, 2.2 L (IQR, 1.7–2.3) at 12 months, and 2.3 L (IQR, 2.0–2.5) at 18 months, with no statistically significant longitudinal change (Friedman *p* = 0.665).

### 3.5. Remission

At 12 months, complete remission was observed in 53/146 patients (36.3%), partial remission in 14/146 (9.6%), and no remission in 79/146 (54.1%). At 18 months, complete remission was observed in 98/146 patients (67.1%), partial remission in 24/146 (16.4%), and no remission in 24/146 (16.4%). In paired analyses, complete remission increased by 30.8 percentage points from 12 to 18 months (95% CI, 23.3 to 38.3; exact McNemar *p* < 0.001), while any remission (complete or partial) increased by 37.7 percentage points (95% CI, 29.8 to 45.5; exact McNemar *p* < 0.001).

At the component level, the proportion free from maintenance OCS increased from 127/146 (87.0%) at 12 months to 136/146 (93.2%) at 18 months; the proportion without persistent symptoms increased from 104/146 (71.2%) to 130/146 (89.0%); and the proportion without exacerbations increased from 132/146 (90.4%) to 140/146 (95.9%). Median ACT increased from 21.0 (IQR, 18.0–22.0) to 22.0 (IQR, 20.0–23.0), whereas median FEV1 remained broadly stable (2.2 L [IQR, 1.7–2.3] at 12 months and 2.3 L [IQR, 2.0–2.5] at 18 months). Because the SANI functional domain is based on longitudinal stability rather than a single numerical threshold, FEV1 is presented as the functional component without imposing a new binary cutoff.

### 3.6. Remission Trajectories

**Figure 1:**
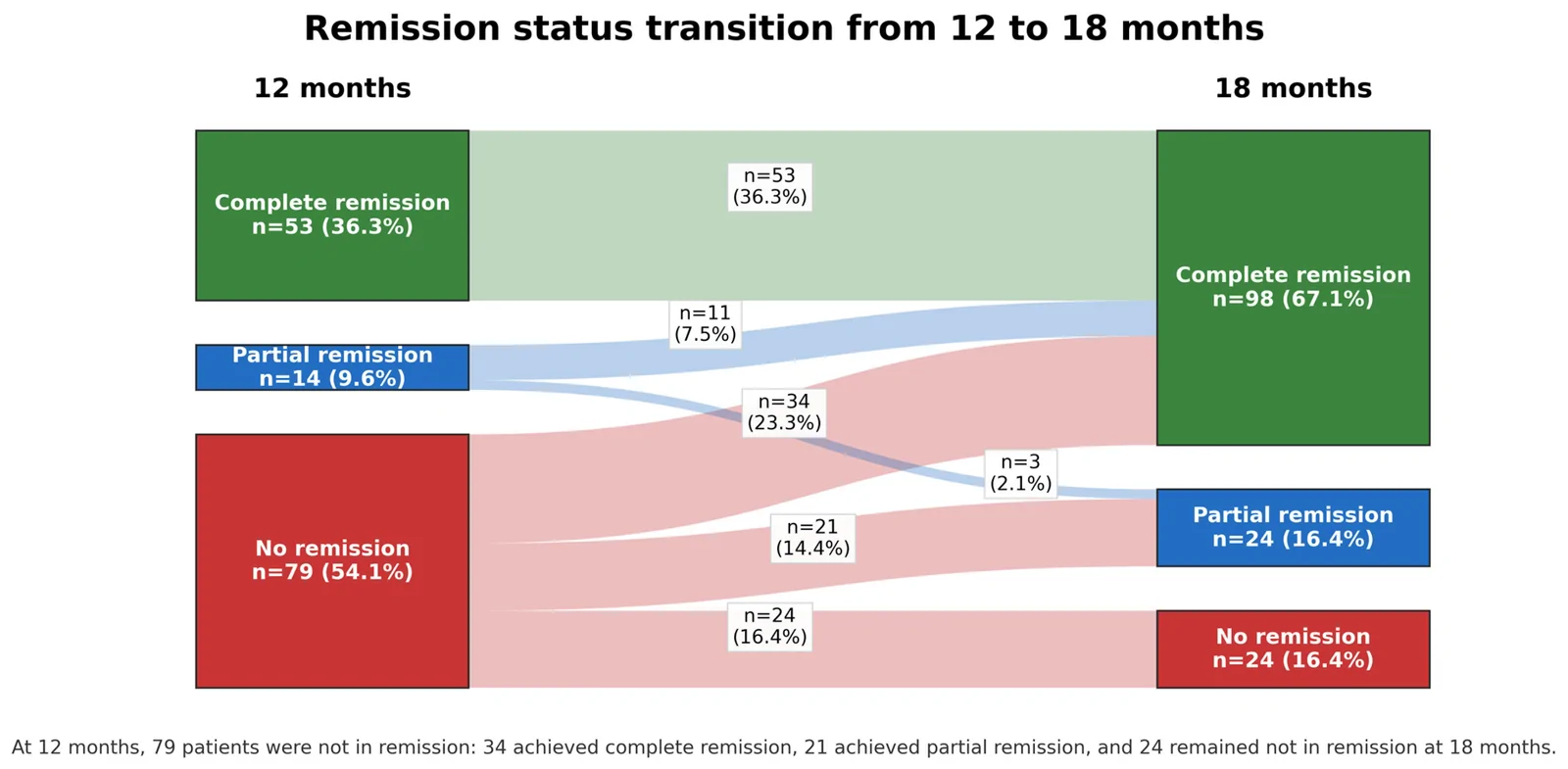
Remission status transitions from 12 to 18 months among patients continuing the same biologic therapy. Complete remission, partial remission, and no remission were defined according to the SANI framework. Flows represent the number and percentage of patients moving between remission categories over time.

Among patients with no remission at 12 months, 34/79 (43.0%) achieved complete remission and 21/79 (26.6%) achieved partial remission at 18 months; the remaining 24/79 (30.4%) remained outside remission. All 53 patients in complete remission at 12 months remained in complete remission at 18 months, and all 14 patients in partial remission either remained partial (3/14) or improved to complete remission (11/14). Thus, no worsening transition was observed in this selected cohort of biologic continuers. The full transition matrix is reported in Table 4 and Figure 1.

### 3.7. Baseline Factors Associated with Remission

At 12 months, baseline oral corticosteroid (OCS) use was strongly associated with remission category. Baseline OCS use was present in 33 of 53 patients (62.3%) achieving complete remission, 10 of 14 (71.4%) achieving partial remission, and 79 of 79 patients (100.0%) with no remission (global *p* < 0.001). ABPA was observed only among patients not achieving complete or partial remission, but this association did not reach conventional statistical significance after the updated classification (5/79 [6.3%] in the no-remission group; *p* = 0.111). No significant association was observed at 12 months for baseline FEV1, ACT, FeNO, total IgE, blood eosinophil count, BMI, CRSwNP, HES/EGPA, sex, or smoking status.

At 18 months, baseline FEV1 differed significantly across remission categories. Patients achieving complete remission had higher baseline FEV1 than those with no remission [median 2.33 L (IQR 2.03–2.71) vs. 1.85 L (IQR 1.62–2.02)], with an intermediate value among patients with partial remission [2.25 L (IQR 1.80–2.36); global *p* = 0.040]. Baseline FEV1 was also positively correlated with ordinal remission category (Spearman rho = 0.36, *p* = 0.014). Baseline OCS use remained associated with lower remission status at 18 months, being present in 75 of 98 patients (76.5%) with complete remission, 23 of 24 (95.8%) with partial remission, and 24 of 24 (100.0%) with no remission (global *p* = 0.004). CRSwNP showed a borderline association with better remission category (*p* = 0.060), whereas HES/EGPA showed a borderline imbalance across groups (*p* = 0.084). Baseline FeNO, total IgE, blood eosinophil count, BMI, baseline ACT, sex, and smoking status were not significantly associated with 18-month remission category (see Table 5).

In exploratory univariable binary analyses, baseline OCS use was inversely associated with both any remission and complete remission at 12 months. At 18 months, higher baseline FEV1 was associated with greater odds of both any remission versus no remission (OR 10.93 per 1 L increase, 95% CI 1.31–91.34; *p* = 0.027) and complete remission versus partial/no remission (OR 5.02 per 1 L increase, 95% CI 1.07–23.68; *p* = 0.041). Baseline OCS use remained inversely associated with remission outcomes, whereas CRSwNP was positively associated with any remission at 18 months (Fisher exact OR 3.59; *p* = 0.022), as shown in Table 6. Because baseline OCS exposure and lung function are closely related to domains used in the SANI remission construct, these associations should be interpreted as exploratory markers of baseline disease burden rather than independent predictors.

## 4. Discussion

In this real-world longitudinal cohort of patients with severe T2-high asthma treated with a single biologic agent, biologic therapy was associated with substantial and progressive improvement across the main domains currently used to assess treatment response and clinical remission. During follow-up, maintenance oral corticosteroid (OCS) use markedly decreased, persistent symptoms became uncommon, asthma control improved substantially, and exacerbations were greatly reduced. These findings are consistent with the established efficacy of biologics in severe asthma and support the shift from single-outcome assessment toward multidimensional treatment targets, including clinical remission [2,4,5,12,13,14,15,16,17,19,20,21,22,23,24,35,36]. The reduction in systemic corticosteroid exposure is particularly relevant because OCS-related toxicity and the risk of pharmacological over-treatment remain important concerns in severe asthma management [6,37].

The most relevant finding of this study is the dynamic evolution of remission status between 12 and 18 months within a selected cohort of patients who remained on an unchanged biologic. Complete remission increased from 36.3% to 67.1%, and any remission increased from 45.9% to 83.6%; paired analyses confirmed that both increases were statistically significant. These data indicate that, among biologic continuers, remission may evolve beyond the conventional 12-month milestone rather than representing a fixed status captured at a single time point.

The transition analysis further reinforces this interpretation. Among patients in partial remission at 12 months, 11/14 (78.6%) progressed to complete remission by 18 months. Among patients not in remission at 12 months, 34/79 (43.0%) achieved complete remission and 21/79 (26.6%) achieved partial remission at 18 months. Importantly, the full transition matrix showed no worsening in remission category. This one-directional pattern is likely influenced by the study design: patients who discontinued or switched biologics because of insufficient response or clinical deterioration were not eligible, enriching the cohort for treatment continuers with acceptable trajectories. It should therefore be interpreted as descriptive of selected continuers, not as evidence that maintaining the same biologic prevents deterioration.

Our results are consistent with the Severe Asthma Network Italy (SANI) remission framework, which distinguishes complete from partial remission and offers a pragmatic tool for real-world severe asthma management [23,24,25]. Partial remission may represent a clinically meaningful intermediate state and, in some patients, a transition toward complete remission. Potential explanations for delayed remission include the time required to reduce OCS exposure, progressive improvement in symptom burden, stabilization of lung function, improved management of comorbidities, and sustained suppression of type 2 inflammatory activity. However, because serial mechanistic biomarker analyses were not available for this study, these explanations remain hypotheses and cannot be established from the present data [7,10,11,14,21,22,32,35,36].

The 12-month complete remission rate observed in our cohort is in line with recent real-world studies reporting clinical remission in approximately one third of biologic-treated patients with severe T2-high asthma, while longer-term cohorts confirm that remission trajectories may vary over time and across populations [19,20,21,22,36,38]. Vervier et al. reported broadly similar 12-month remission proportions across omalizumab, mepolizumab, and benralizumab in a real-world cohort [19]. In our cohort, baseline profiles differed substantially by prescribed biologic, particularly for CRSwNP and OCS exposure. We therefore present biologic stratification as clinically informative descriptive context rather than as a head-to-head efficacy comparison, because treatment allocation was not randomized and was strongly phenotype- and comorbidity-driven.

Baseline OCS use was associated with a lower probability of remission. Maintenance OCS identifies patients with greater baseline disease burden and corticosteroid dependence; however, OCS exposure is also closely related to the mandatory OCS-free remission domain. The observed association is therefore partly structural and should not be interpreted as an independent causal predictor. It is better viewed as a hypothesis-generating marker of baseline severity and of the greater therapeutic distance required before stringent remission criteria can be fulfilled.

Better preserved baseline lung function was associated with more favorable remission status at 18 months. As with OCS exposure, this finding requires caution because lung-function stability is itself a SANI remission component. Higher baseline FEV1 may indicate less fixed airflow limitation or structural airway damage, but this mechanistic interpretation remains hypothetical in the absence of direct measurements of airway remodeling. The association should therefore be considered exploratory and requires validation in larger cohorts.

The role of comorbidities deserves consideration. ABPA was observed only among patients not achieving remission at 12 months, although the small number of cases limits interpretation [28]. HES/EGPA may complicate asthma-specific remission assessment, and CRSwNP was more frequent among patients achieving remission at 18 months. These patterns may reflect differences in phenotype, comorbidity burden, and biologic selection, but they cannot establish mechanism or treatment superiority in this observational cohort. In particular, any explanation based on differential suppression of type 2 inflammation should be regarded as hypothesis-generating because serial mechanistic biomarker data were not analyzed [7,14,18,39].

No significant association was observed between remission and baseline FeNO, total IgE, blood eosinophils, BMI, sex, smoking status, or baseline ACT. These findings should be interpreted cautiously. All patients had severe T2-high asthma and were selected for biologic therapy according to clinical and biomarker-based criteria; therefore, the ability of individual biomarkers to further discriminate remission probability may have been limited. Nevertheless, blood eosinophils, FeNO, IgE, and clinical traits remain essential for phenotyping, biologic eligibility, and treatment monitoring in severe asthma [14,29,32,35]. ACT also remains a validated and clinically meaningful tool for assessing asthma control, and exacerbation outcomes should be interpreted according to standardized definitions [33,34]. The absence of a sex-related signal in our cohort should not exclude the relevance of sex-based mechanisms in asthma pathophysiology and treatment response, which may require larger and specifically designed studies [3]. The lack of BMI and smoking-status signals in our cohort is consistent with real-world evidence showing preserved biologic effectiveness in obese patients and former smokers, although study populations and biologics differ [38,40].

This study has several strengths, including its real-world design, the use of a clinically applicable remission definition, and the longitudinal assessment of remission transitions in patients treated with a single biologic agent. By focusing on patients who continued a single biologic, the study specifically describes remission trajectories in a clinically relevant subgroup of biologic-treated severe asthma patients. This design allows longitudinal assessment of patients who remained on the same treatment, while avoiding attribution of later outcomes to multiple biologic exposures.

Several limitations should, however, be acknowledged. First, the retrospective single-center design limits generalizability and precludes causal inference. The principal limitation is selection bias: by requiring at least 18 months of follow-up on the same biologic and excluding every patient who discontinued or switched, the cohort necessarily omits patients with inadequate response, intolerance, or clinical deterioration leading to a treatment change. This creates completer/survivor bias and may partly explain the absence of worsening transitions. Accordingly, the findings describe remission trajectories among selected biologic continuers and must not be generalized to all patients who fail to achieve remission at 12 months. Second, biologic subgroups were clinically heterogeneous and treatment allocation was confounded by indication; tezepelumab was represented by only two patients. Third, the limited number of 18-month non-remitters, sparse cells, and separation for baseline OCS exposure prevented a stable multivariable model adjusting simultaneously for baseline severity, biologic type, and remission-related variables; residual confounding therefore remains. Concomitant high-dose ICS/LABA plus LAMA therapy was essentially uniform and did not provide meaningful variation for adjustment. Fourth, selected spirometric and biomarker measurements were missing and were analyzed using available cases without imputation, although remission classification and the principal categorical remission components were complete at both landmarks. Finally, serial mechanistic biomarker analyses were not available, so proposed biological explanations for delayed remission remain hypotheses.

Asthma remission remains a variably defined construct across studies [41]. In the present study, the SANI framework included stable lung function as a core criterion but did not mandate a single numerical FEV1 threshold; therefore, lung-function stability was assessed longitudinally according to the SANI definition rather than by imposing an additional cutoff [23,24].

In conclusion, among patients with severe T2-high asthma selected for continuing an unchanged biologic for at least 18 months, remission status was more favorable at 18 months than at 12 months, and many 12-month non-remitters subsequently fulfilled partial or complete remission criteria. These findings support longitudinal multidomain assessment beyond a single 12-month snapshot in biologic continuers. However, they do not establish that continuation is preferable to switching and should not be generalized to all 12-month non-remitters. Prospective multicenter studies including both continuers and switchers are needed to define optimal treatment decisions after incomplete remission.

## Figures and Tables

**Table 1:** Baseline characteristics of the enrolled cohort.

Characteristic	Overall Cohort (N = 146)
Age, years	59.9 ± 13.4; 61.0 (52.0–70.0)
Female sex, N (%)	94 (64.4)
Male sex, N (%)	52 (35.6)
BMI, kg/m^2^	24.2 ± 2.3; 23.8 (22.3–26.3)
Normal weight, BMI < 25 kg/m^2^	91 (62.3)
Overweight, BMI 25–29.9 kg/m^2^	55 (37.7)
Obesity, BMI ≥ 30 kg/m^2^	0 (0.0)
Never-smoker, N (%)	101 (69.2)
Former smoker, N (%)	31 (21.2)
Current smoker, N (%)	14 (9.6)
Any recorded comorbidity, N (%)	69 (47.3)
CRSwNP, N (%)	55 (37.7)
HES/EGPA, N (%)	9 (6.2)
ABPA, N (%)	5 (3.4)
Eosinophilic esophagitis, N (%)	2 (1.4)
Atopic dermatitis, N (%)	2 (1.4)
COPD/ACOS, N (%)	3 (2.1)
CSU, N (%)	1 (0.7)
IgG4-related disease, N (%)	1 (0.7)
T2-high phenotype, N (%)	146 (100.0)
T2-low/non-T2 phenotype, N (%)	0 (0.0)
Maintenance OCS use before biologic initiation, N (%)	122 (83.6)
Asthma symptoms before biologic initiation, N (%)	146 (100.0)
Baseline ACT score	12.4 ± 3.9; 12.0 (9.0–16.0)
Baseline FEV1, L	2.2 ± 0.5; 2.3 (2.0–2.5)
Baseline FeNO, ppb	47.4 ± 7.3; 47.4 (40.7–53.4)
Baseline total IgE, IU/mL	256.0 ± 117.3; 257.4 (155.9–342.4)
Baseline blood eosinophils, cells/uL	318 ± 212; 288 (206–372)
SNOT-22	38.0 ± 7.5; 39.3 (31.3–44.4)
NPS	1.1 ± 1.5; 0.0 (0.0–2.2)

Note. Values are mean ± SD; median (IQR). Abbreviations: ABPA, allergic bronchopulmonary aspergillosis; ACOS, asthma–COPD overlap syndrome; ACT, Asthma Control Test; BMI, body mass index; COPD, chronic obstructive pulmonary disease; CRSwNP, chronic rhinosinusitis with nasal polyps; CSU, chronic spontaneous urticaria; EGPA, eosinophilic granulomatosis with polyangiitis; FeNO, fractional exhaled nitric oxide; FEV1, forced expiratory volume in 1 s; HES, hypereosinophilic syndrome; IgE, immunoglobulin E; IgG4, immunoglobulin G4; NPS, Nasal polyp score; OCS, oral corticosteroids; SNOT-22, 22-item Sinonasal Outcome Test; T2, type 2.

**Table 2:** Biologic therapies and baseline profiles stratified by prescribed biologic.

Biologic	Patients, n (%)	Female, n (%)	Age, Median (IQR), Years	Baseline OCS, n (%)	CRSwNP, n (%)	Baseline FeNO, Median (IQR), ppb	Baseline Eosinophils, Median (IQR), Cells/uL
Omalizumab	41 (28.1)	29 (70.7)	62.0 (52.8–70.0)	35 (85.4)	7 (17.1)	48.4 (41.5–53.1)	287 (194–376)
Benralizumab	34 (23.3)	22 (64.7)	60.0 (52.0–71.0)	25 (73.5)	12 (35.3)	47.6 (43.5–54.5)	292 (220–354)
Mepolizumab	28 (19.2)	20 (71.4)	61.0 (57.2–70.0)	26 (92.9)	5 (17.9)	49.6 (40.1–56.5)	305 (212–388)
Dupilumab	41 (28.1)	22 (53.7)	59.0 (47.0–69.5)	34 (82.9)	31 (75.6)	45.4 (40.3–51.6)	274 (192–366)
Tezepelumab	2 (1.4)	1 (50.0)	47.0 (41.5–52.5)	2 (100.0)	0 (0.0)	46.3 (46.1–46.6)	330 (275–384)

**Table 3:** Longitudinal outcomes of the enrolled cohort.

Outcome	Baseline	6 Months	12 Months	18 Months	Global *p* Value
OCS use, n (%)	122 (83.6)	66 (45.2)	19 (13.0)	10 (6.8)	<0.001
Symptoms, n (%)	146 (100.0)	86 (58.9)	42 (28.8)	16 (11.0)	<0.001
ACT score, median (IQR); n	12.0 (9.0–16.0)	16.0 (11.2–21.0)	21.0 (18.0–22.0)	22.0 (20.0–23.0)	<0.001
FEV1, L, median (IQR); n	2.3 (2.0–2.5)	2.1 (1.4–2.3)	2.2 (1.7–2.3)	2.3 (2.0–2.5)	0.665
Patients with ≥2 exacerbations in the previous year at baseline or ≥1 exacerbation during follow-up, n (%)	146 (100.0)	46 (31.5)	14 (9.6)	6 (4.1)	<0.001

**Table 4:** Remission-component indicators and complete 12-to-18-month remission transition matrix.

Panel A. Remission-Component Indicators				
Measure	12 Months	18 Months	Paired/Transition Information	
Free from maintenance OCS	127/146 (87.0%)	136/146 (93.2%)	+6.2 percentage points	
No persistent symptoms	104/146 (71.2%)	130/146 (89.0%)	+17.8 percentage points	
No exacerbation	132/146 (90.4%)	140/146 (95.9%)	+5.5 percentage points	
ACT score, median (IQR)	21.0 (18.0–22.0)	22.0 (20.0–23.0)	Improved asthma control	
FEV1, L, median (IQR)	2.2 (1.7–2.3)	2.3 (2.0–2.5)	Broadly stable	
**Panel B. Complete 12-to-18-month remission transition matrix**				
12-month status	18-month complete	18-month partial	18-month no remission	Row total
Complete remission	53	0	0	53
Partial remission	11	3	0	14
No remission	34	21	24	79
Total	98	24	24	146

Note. Component indicators are descriptive and use the definitions applied in the main longitudinal analysis. FEV1 is shown as a continuous functional measure because the SANI framework defines the functional domain by longitudinal stability rather than by an additional fixed numerical cutoff. The transition matrix includes all 146 patients.

**Table 5:** Baseline factors associated with remission status.

Time Point	Baseline Factor	Complete Remission	Partial Remission	No Remission	*p* Value
12 months	Baseline OCS use	33/53 (62.3%)	10/14 (71.4%)	79/79 (100.0%)	<0.001
12 months	ABPA	0/53 (0.0%)	0/14 (0.0%)	5/79 (6.3%)	0.111
18 months	Baseline FEV1, L, median (IQR)	2.33 (2.03–2.71)	2.25 (1.80–2.36)	1.85 (1.62–2.02)	0.040
18 months	Baseline OCS use	75/98 (76.5%)	23/24 (95.8%)	24/24 (100.0%)	0.004
18 months	CRSwNP	42/98 (42.9%)	9/24 (37.5%)	4/24 (16.7%)	0.060
18 months	HES/EGPA	3/98 (3.1%)	3/24 (12.5%)	3/24 (12.5%)	0.084

Note. Continuous variables are median (IQR); n. Categorical variables are n (%). Global *p* values refer to comparisons across complete remission, partial remission, and no remission.

**Table 6:** Exploratory univariable binary models.

Outcome	Predictor	Effect Estimate	*p* Value
Any remission vs. no remission at 12 months	Baseline OCS use	Complete separation; Fisher exact *p* value reported	<0.001
Complete remission vs. partial/no remission at 12 months	Baseline OCS use	OR 0.07 (Fisher exact)	<0.001
Any remission vs. no remission at 18 months	Baseline FEV1 [L]	10.93 (1.31–91.34)	0.027
Any remission vs. no remission at 18 months	Baseline OCS use	Complete separation; Fisher exact *p* value reported	0.014
Any remission vs. no remission at 18 months	CRSwNP	OR 3.59 (Fisher exact)	0.022
Complete remission vs. partial/no remission at 18 months	Baseline FEV1 [L]	5.02 (1.07–23.68)	0.041
Complete remission vs. partial/no remission at 18 months	Baseline OCS use	OR 0.07 (Fisher exact)	<0.001

Note. Univariable models only. For categorical predictors with sparse cells or complete separation, standard logistic regression estimates were unstable; therefore, exact *p* values or descriptive summaries are reported. These results should be interpreted as exploratory.

## Data Availability

The data presented in this study are available from the corresponding author upon reasonable request. The data are not publicly available due to privacy and ethical restrictions related to clinical patient-level data.

## References

[1] Global Initiative for Asthma Global Strategy for Asthma Management and Prevention. 2025 Update. https://ginasthma.org/wp-content/uploads/2025/11/GINA-2025-Update-25_11_08-WMS.pdf.

[2] Gyawali B., Georas S.N., Khurana S. (2025). Biologics in severe asthma: A state-of-the-art review. Eur. Respir. Rev..

[3] Borrelli R., Brussino L., Lo Sardo L., Quinternetto A., Vitali I., Bagnasco D., Boem M., Corradi F., Badiu I., Negrini S. (2025). Sex-Based Differences in Asthma: Pathophysiology, Hormonal Influence, and Genetic Mechanisms. Int. J. Mol. Sci..

[4] Volmer T., Effenberger T., Trautner C., Buhl R. (2018). Consequences of long-term oral corticosteroid therapy and its side-effects in severe asthma in adults: A focused review of the impact data in the literature. Eur. Respir. J..

[5] Lupia C., Marchi G., Chiarella E., Piazzetta G.L., Lobello N., Crimi C., Poto R., Maglio A., Vatrella A., Pelaia G. (2026). Predictive Factors And Treatments Associated with Clinical Remission in Severe Eosinophilic Asthma. J. Inflamm. Res..

[6] Sullivan P.W., Ghushchyan V.H., Globe G., Schatz M. (2018). Oral corticosteroid exposure and adverse effects in asthmatic patients. J. Allergy Clin. Immunol..

[7] Matucci A., Micheletto C., Vultaggio A. (2023). Severe Asthma and Biologics: Managing Complex Patients. J. Investig. Allergol. Clin. Immunol..

[8] Suruki R.Y., Daugherty J.B., Boudiaf N., Albers F.C. (2017). The frequency of asthma exacerbations and healthcare utilization in patients with asthma from the UK and USA. BMC Pulm. Med..

[9] Guarnieri M., Balmes J.R. (2014). Outdoor air pollution and asthma. Lancet.

[10] Hough K.P., Curtiss M.L., Blain T.J., Liu R.M., Trevor J., Deshane J.S., Thannickal V.J. (2020). Airway Remodeling in Asthma. Front. Med..

[11] Kardas G., Kuna P., Panek M. (2020). Biological Therapies of Severe Asthma and Their Possible Effects on Airway Remodeling. Front. Immunol..

[12] Gunaydin F.E., Marcassa G., Olivieri B., Senna G., Caminati M. (2026). Is Asthma Remission a New Achievable Goal? A Review of the Current Drug-Related Evidence and Predictors. Allergy.

[13] Menzies-Gow A., Bafadhel M., Busse W.W., Casale T.B., Kocks J.W.H., Pavord I.D., Szefler S.J., Woodruff P.G., de Giorgio-Miller A., Trudo F. (2020). An expert consensus framework for asthma remission as a treatment goal. J. Allergy Clin. Immunol..

[14] Pastore D., Lupia C., Chiarella E., Piazzetta G.L., Mazza G., Neri G., Bruni A., Longhini F., Garofalo E., Pelaia G. (2026). Severe Eosinophilic Asthma: From Immunopathology to Pharmacological Treatment. J. Clin. Med..

[15] Brusselle G.G., Koppelman G.H. (2022). Biologic therapies for severe asthma. N. Engl. J. Med..

[16] Bagnasco D., Bondi B., Brussino L., Nicola S., Cameli P., Tiotiu A., Guida G., Gollinucci C., Visca D., Spanevello A. (2025). Dupilumab Effectiveness in Patients with Severe Allergic Asthma Non-Responsive to Omalizumab. J. Pers. Med..

[17] Caminati M., Maule M., Benoni R., Bagnasco D., Beghe B., Braido F., Brussino L., Cameli P., Candeliere M.G., Carpagnano G.E. (2024). Dupilumab Efficacy on Asthma Functional, Inflammatory, and Patient-Reported Outcomes across Different Disease Phenotypes and Severity: A Real-Life Perspective. Biomedicines.

[18] Caminati M., Mastrototaro A., Maule M., Schiappoli M., Vaia R., Zurlo M., Bini F., Brussino L., D’Amato M., Marra A.M. (2026). Upper and lower airways response to tezepelumab in asthma patients with/without comorbid nasal polyposis: A 6-month real-life perspective. Rhinology.

[19] Vervier J., Sabbe M., Louis F., Moermans C., Guissard F., Sanchez C., Paulus V., Henket M., Philippe G., Beijnink C.W.H. (2026). Clinical Remission in Severe T2-High Asthma in Real Life After Anti-IgE, Anti-IL-5 and Anti-IL5R: A Potential Role for CRP as a Biomarker. Clin. Transl. Allergy.

[20] Mărginean C., Safta A.C., Huțanu D., Budin C.E., Ianoși M.B., Jimborean G., Ianoși E.S. (2026). Long-Term Clinical and Biological Outcomes of Biologic Therapy in Severe Asthma: 24-Month Real-World Cohort Study from Romania. J. Clin. Med..

[21] Eger K., Kroes J.A., Ten Brinke A., Bel E.H. (2021). Long-Term Therapy Response to Anti-IL-5 Biologics in Severe Asthma-A Real-Life Evaluation. J. Allergy Clin. Immunol. Pract..

[22] McDowell P.J., McDowell R., Busby J., Eastwood M.C., Patel P.H., Jackson D.J., Mansur A., Patel M., Burhan H., Doe S. (2023). Clinical remission in severe asthma with biologic therapy: An analysis from the UK Severe Asthma Registry. Eur. Respir. J..

[23] Canonica G.W., Blasi F., Carpagnano G.E., Guida G., Heffler E., Paggiaro P., on behalf of SANI (2023). Severe Asthma Network Italy Definition of Clinical Remission in Severe Asthma: A Delphi Consensus. J. Allergy Clin. Immunol. Pract..

[24] Canonica G.W., Bagnasco D., Bondi B., Varricchi G., Paoletti G., Blasi F., Paggiaro P., Braido F., SANI study group (2024). SANI clinical remission definition: A useful tool in severe asthma management. J. Asthma.

[25] Ledda A.G., Costanzo G., Canalis S., Puci M.V., Sambugaro G., Taurisano G., Piano E., Bullita M., Sotgiu G., Firinu D. (2026). Evaluation of the concordance of asthma remission definitions according to international guidelines in a cohort of patients treated with monoclonal antibodies for severe asthma. Front. Pharmacol..

[26] Zheng Y., Li Y.C., Li S.J., Xu M., Hu J.Q., Tian W.Q., Chen S.W., Li X.H., He Y., Lu G. (2026). Patterns and Clinical Efficacy of Biologics Switching in Patients with Severe Asthma: A Systematic Review and Meta-Analysis. Allergy.

[27] Sin D.D., Miravitlles M., Mannino D.M., Soriano J.B., Price D., Celli B.R., Leung J.M., Nakano Y., Park H.Y., Wark P.A. (2016). What is asthma-COPD overlap syndrome? Towards a consensus definition from a round table discussion. Eur. Respir. J..

[28] Agarwal R., Sehgal I.S., Dhooria S., Aggarwal A.N. (2016). Developments in the diagnosis and treatment of allergic bronchopulmonary aspergillosis. Expert Rev. Respir. Med..

[29] Korevaar D.A., Westerhof G.A., Wang J., Cohen J.F., Spijker R., Sterk P.J., Bel E.H., Bossuyt P.M. (2015). Diagnostic accuracy of minimally invasive markers for detection of airway eosinophilia in asthma: A systematic review and meta-analysis. Lancet Respir. Med..

[30] American Thoracic Society, European Respiratory Society (2005). ATS/ERS recommendations for standardized procedures for the online and offline measurement of exhaled lower respiratory nitric oxide and nasal nitric oxide. Am. J. Respir. Crit. Care Med..

[31] Dweik R.A., Boggs P.B., Erzurum S.C., Irvin C.G., Leigh M.W., Lundberg J.O., Olin A.C., Plummer A.L., Taylor D.R., American Thoracic Society Committee on Interpretation of Exhaled Nitric Oxide Levels (FENO) for Clinical Applications (2011). An official ATS clinical practice guideline: Interpretation of exhaled nitric oxide levels (FENO) for clinical applications. Am. J. Respir. Crit. Care Med..

[32] Bourdin A., Brusselle G., Couillard S., Fajt M.L., Heaney L.G., Israel E., McDowell P.J., Menzies-Gow A., Martin N., Mitchell P.D. (2024). Phenotyping of Severe Asthma in the Era of Broad-Acting Anti-Asthma Biologics. J. Allergy Clin. Immunol. Pract..

[33] Reddel H.K., Taylor D.R., Bateman E.D., Boulet L.P., Boushey H.A., Busse W.W., Casale T.B., Chanez P., Enright P.L., Gibson P.G. (2009). An official American Thoracic Society/European Respiratory Society statement: Asthma control and exacerbations: Standardizing endpoints for clinical asthma trials and clinical practice. Am. J. Respir. Crit. Care Med..

[34] Nathan R.A., Sorkness C.A., Kosinski M., Schatz M., Li J.T., Marcus P., Murray J.J., Pendergraft T.B. (2004). Development of the Asthma Control Test: A survey for assessing asthma control. J. Allergy Clin. Immunol..

[35] FitzGerald J.M., Bleecker E.R., Nair P., Korn S., Ohta K., Lommatzsch M., Ferguson G.T., Busse W.W., Barker P., Sproule S. (2016). Benralizumab, an anti-interleukin-5 receptor alpha monoclonal antibody, as add-on treatment for patients with severe, uncontrolled, eosinophilic asthma (CALIMA): A randomised, double-blind, placebo-controlled phase 3 trial. Lancet.

[36] Ortega H.G., Liu M.C., Pavord I.D., Brusselle G.G., FitzGerald J.M., Chetta A., Humbert M., Katz L.E., Keene O.N., Yancey S.W. (2014). Mepolizumab Treatment in Patients with Severe Eosinophilic Asthma. N. Engl. J. Med..

[37] Bleecker E.R., Menzies-Gow A.N., Price D.B., Bourdin A., Sweet S., Martin A.L., Alacqua M., Tran T.N. (2020). Systematic Literature Review of Systemic Corticosteroid Use for Asthma Management. Am. J. Respir. Crit. Care Med..

[38] Gonem S., Redmond C., Busby J., Patel P., Jackson D.J., Mansur A.H., Pfeffer P., Pantin T., Brown T., Patel M. (2025). Effects of obesity on response to asthma biologic treatment: Longitudinal data from the United Kingdom Severe Asthma Registry. J. Allergy Clin. Immunol. Pract..

[39] Li S.H., Foster Nehme K., Moshkovich A., Suh L., Pawlowski A., Ali Y., Patel G.B., Kuang F.L., Peters A.T. (2025). Eosinophilia and Adverse Effects of Dupilumab for Respiratory Indications: A Real-World Setting. J. Allergy Clin. Immunol. Pract..

[40] Baastrup Soendergaard M., Hansen S., Bjerrum A.S., von Bülow A., Haakansson K.E.J., Hilberg O., Ingebrigtsen T.S., Johnsen C.R., Lock-Johansson S., Makowska Rasmussen L. (2024). Tobacco Exposure and Efficacy of Biologic Therapy in Patients with Severe Asthma: A Nationwide Study from the Danish Severe Asthma Register. J. Allergy Clin. Immunol. Pract..

[41] Thomas D., McDonald V.M., Pavord I.D., Gibson P.G. (2022). Asthma remission: What is it and how can it be achieved?. Eur. Respir. J..

